# An Approach to Identifying and Quantifying Bias in Biomedical Data

**Published:** 2023

**Authors:** M. Clara De Paolis Kaluza, Shantanu Jain, Predrag Radivojac

**Affiliations:** Northeastern University, Boston, MA 02115, U.S.A.

**Keywords:** Bias detection, bias estimation, semi-supervised learning

## Abstract

Data biases are a known impediment to the development of trustworthy machine learning models and their application to many biomedical problems. When biased data is suspected, the assumption that the labeled data is representative of the population must be relaxed and methods that exploit a typically representative unlabeled data must be developed. To mitigate the adverse effects of unrepresentative data, we consider a binary semi-supervised setting and focus on identifying whether the labeled data is biased and to what extent. We assume that the class-conditional distributions were generated by a family of component distributions represented at different proportions in labeled and unlabeled data. We also assume that the training data can be transformed to and subsequently modeled by a nested mixture of multivariate Gaussian distributions. We then develop a multi-sample expectation-maximization algorithm that learns all individual and shared parameters of the model from the combined data. Using these parameters, we develop a statistical test for the presence of the general form of bias in labeled data and estimate the level of this bias by computing the distance between corresponding class-conditional distributions in labeled and unlabeled data. We first study the new methods on synthetic data to understand their behavior and then apply them to real-world biomedical data to provide evidence that the bias estimation procedure is both possible and effective.

## 1. Introduction

The development and application of machine learning methods have become commonplace in biomedical sciences and have the potential to transform clinical care.^1, 2^ Many of those predictive modeling approaches take place in a binary semi-supervised setting; that is, where the prediction outcome is dichotomized and the available data for training and evaluation contains samples of labeled and unlabeled examples. One such scenario is the prediction of the effect of genomic variants as pathogenic or benign, where labeled data contains pathogenic (positive) and benign (negative) variants from databases such as ClinVar^3^ and the unlabeled data is often a large reference set of observed variants such as gnomAD.^4^

A traditional approach in semi-supervised learning is to assume that the labeled data is representative of unlabeled data, thus requiring little sophistication during model development, model selection, and performance evaluation. However, a distinguishing feature of real biomedical data is that the labeled examples may not be representative of the unlabeled data; that is, the labeled data may be biased.^5^ Data biases can have adverse effects on the ability of models to be optimized for the unlabeled data at hand and can also lead to poor estimation of a classifier’s performance on a reference distribution.^6^ More generally, biased data presents an obstacle to the development of trustworthy methods that are necessary for the societal acceptance of machine learning-based predictive technologies.^7, 8^

Learning under sample selection bias is a well-known problem.^9^ Early approaches relaxed the assumption of fully representative data by assuming the same class-conditional distributions in labeled and unlabeled data, thus reducing the problem of posterior estimation to estimation of class priors in unlabeled data.^10, 11^ Other approaches consider situations where at least one class-conditional distribution from which the labeled data is generated is representative of its unlabeled counterpart.^12–15^ While such methods have advanced the treatment of sample selection bias, we are not aware of methods that can identify whether and to what extent labeled data differs from unlabeled data for a general form of bias.

The objective of this work is to develop a statistical test for identifying biased labeled data while simultaneously quantifying the level of bias. We assume that the real-world data can be transformed and subsequently modeled using nested mixtures of multivariate Gaussian distributions; that is, with both positive and negative samples being Gaussian mixtures themselves. We then model these class-conditional distributions in both labeled and unlabeled data by the shared underlying component distributions, but permit the proportions at which the data is sampled from those component distributions to differ between labeled and unlabeled data. We finally develop an expectation-maximization (EM) algorithm that learns both individual and shared parameters from the combined data which allows us to identify and quantify bias. Our experiments on synthetic and real-world data demonstrate the ability of this procedure to detect bias and provide useful information to data scientists in their workflows.

## 2. Problem Formulation

We consider the binary classification problem where input features $x\in {\mathbb{R}}^{D}$ are used to predict class label *y* ∈ 𝒴 = {−, +}, where + and − represent the positive and negative class, respectively. Let *p*(*x*, *y*) be the unknown joint distribution that governs how *x* appears in nature or in a target population of interest and its relationship with *y*. We refer to *p*(*x*, *y*) as the unbiased distribution, where we expect a classifier to perform optimally. Let *f*_+_(*x*) = *p*(*x*|*y* = +) and *f*_−_(*x*) = *p*(*x*|*y* = −) denote the positive and negative class-conditional distributions, respectively. Let *f*(*x*) = *p*(*x*) denote the marginal distribution over *x* and *α* = *p*(*y* = +) be the probability that a random point from *p*(*x*, *y*) is positive, the class prior for the positive class. It can be shown that *f* is a mixture distribution with components *f*_+_ and *f*_−_ and mixing proportions *α* and 1 − *α*, respectively; i.e.,

(1) $$f(x)=\alpha f_{+}(x)+(1-\alpha )f_{-}(x).$$

**Fig. 1:**
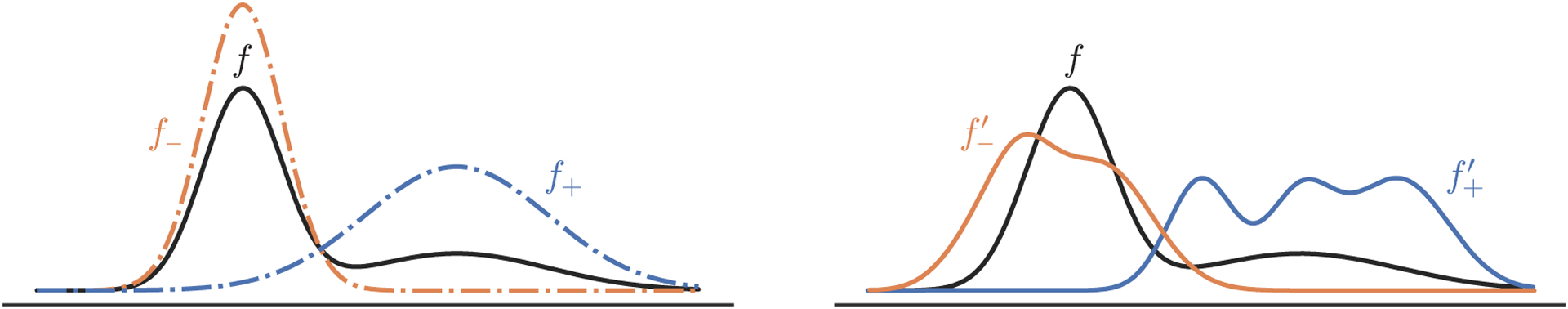
An illustration of bias in labeled data. Left: unbiased (unobserved, dash-dotted lines) distributions of positive (*f*_+_) and negative (*f*_−_) classes that comprise the (observed, solid line) unbiased mixture distribution *f* = *αf*_+_ + (1 − *α*)*f*_−_, drawn here with *α* = 0.3. Right: the same unbiased observed mixture *f* together with biased observed distributions of positive ($f_{+}^{\prime }$) and negative ($f_{-}^{\prime }$) classes. The objective of this work is to use datasets from (*f*, $f_{+}^{\prime }$, $f_{-}^{\prime }$) to estimate the existence and extent of the differences between *f*_+_ and $f_{+}^{\prime }$ and between *f*_−_ and $f_{-}^{\prime }$.

Let *L*^+^ and *L*^−^ represent sets of positive and negative labeled examples, respectively and *U* represent a set of unlabeled examples, available for training. Though we observe examples drawn randomly from *f*(*x*) in *U*, unlike the standard classification setting, we might not observe labeled examples drawn randomly from *f*_+_(*x*) and *f*_−_(*x*). Instead *L*^+^ and *L*^−^ are drawn from potentially biased class-conditional distributions $f_{+}^{\prime }(x)$ and $f_{-}^{\prime }(x)$, respectively (Fig. 1). We use the term bias here in a purely statistical sense; the labeled positives and negatives in the observed data are systemically different from those in the unlabeled data such that they cannot be interpreted to be drawn i.i.d. from the same distribution. In this work, we are interested in detecting and quantifying the extent to which the examples in *L*^+^ and *L*^−^ differ from the positives and negatives in *U*, without the knowledge of the class labels in *U*.

### 2.1. Assumptions

If $f_{+}^{\prime }(x)$ and $f_{-}^{\prime }(x)$ are arbitrarily different from *f*_+_(*x*) and *f*_−_(*x*), respectively, detecting and quantifying the bias is an intractable problem. Fortunately, for most practical settings the biased and unbiased distributions are related. In this work, we employ a (G)aussian (c)omponent-based “(m)ixing (b)ias” assumption (MB-GC),^16^ relating the biased and unbiased distributions. Formally, we assume both *f*_+_(*x*) and $f_{+}^{\prime }(x)$ can be expressed as mixtures with the same *K*^+^ shared Gaussian component distributions, but with differing mixing proportions. *f*_−_(*x*) and $f_{-}^{\prime }(x)$ are assumed to be related in the same manner with *K*^−^ shared Gaussian components. Mathematically,

(MB-GC) $$f_{*}(x)=\sum_{k\in {𝒦}^{*}}w_{k}^{*}\phi_{k}^{*}(x)\;\;\text{and}\;\;f_{*}^{\prime }(x)=\sum_{k\in {𝒦}^{*}}v_{k}^{*}\phi_{k}^{*}(x),$$

where * is a placeholder for + or −; 𝒦* = {1, 2, …, K*}; $w^{*}={\left[w_{k}^{*}\right]}_{k\in {𝒦}^{*}}$ and $v^{*}={\left[v_{k}^{*}\right]}_{k\in {𝒦}^{*}}$ are probability vectors; i.e., $w_{k}^{*}$, $v_{k}^{*}\geq 0$, $\sum_{j\in {𝒦}^{*}}w_{j}^{*}=1$ and $\sum_{j\in {𝒦}^{*}}v_{j}^{*}=1$; and $\phi_{k}^{*}(x)=\phi \left(x;\mu_{k}^{*},\Sigma_{k}^{*}\right)$ is the *D*-dimensional Gaussian density function with mean $\mu_{k}^{*}$ and covariance $\Sigma_{k}^{*}$. We use the shorthand $\mu^{*}={\left\{\mu_{k}^{*}\right\}}_{k\in {𝒦}^{*}}$ and $\Sigma^{*}={\left\{\Sigma_{k}^{*}\right\}}_{k\in {𝒦}^{*}}$ to group the parameters.

It is important to mention that a parametric approximation of the distributions becomes a universal nonparametric approximator as *K*^+^, *K*^−^ → ∞.^17^ However, picking a large number of components may lead to a complex model prone to overfitting and identifiability issues. We therefore restrict ourselves to a relatively small number of components, up to eight, in each class-conditional representation, as in the parametric paradigm.

Since Gaussian mixture models are effective up to a moderate number dimensions, for high-dimensional data, we employ the MB-GC assumption after dimensionality reduction. Conceptually, we interpret the input feature $x\in {\mathbb{R}}^{D}$ as a low-dimensional representation of *D*_*r*_-dimensional raw features (*D*_*r*_ > *D*) in such cases. It is conceivable that neither the raw features nor the dimensionality-reduced features appear exactly as Gaussian mixtures, especially with a small number of components. In spite of this limitation, we argue that the modern representation learning approaches^18, 19^ can be used to learn embeddings that do satisfy that property, potentially making our assumptions and methods even more effective.

### 2.2. Quantifying Bias

**Fig. 2:**
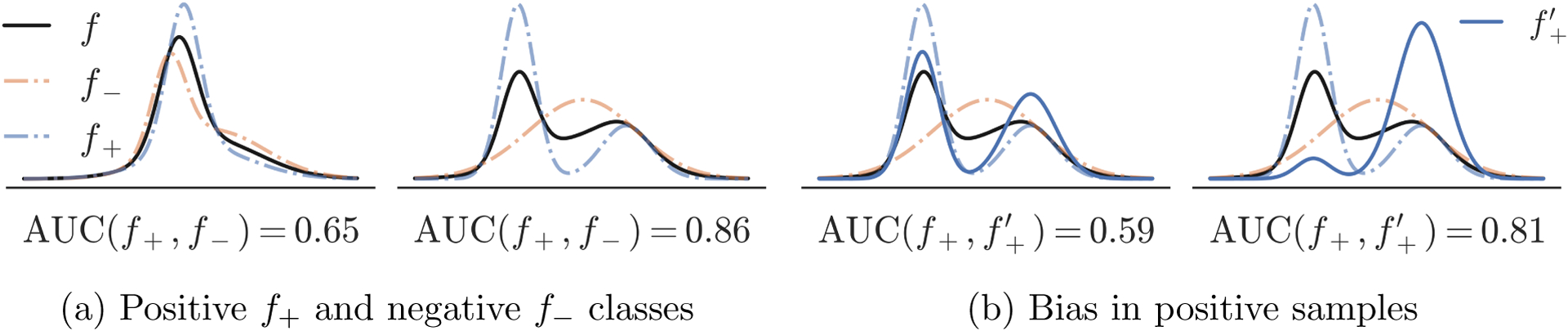
Synthetic data in one dimension. Examples of (a) low and high AUC(*f*_+_, *f*_−_) and (b) low and high bias AUC(*f*_+_, $f_{+}^{\prime }$). Unlabeled mixtures *f* shown here with *α* = 0.5 in all cases.

Although various distance measures can be used,^20^ we quantify the bias between *f*_+_ and $f_{+}^{\prime }$ as the area under the ROC curve (AUC) of an optimal binary classifer, or a score function $s:{\mathbb{R}}^{D}\to \mathbb{R}$, between them. Based on the probabilistic interpretation of AUC,^21^ it is the probability that a randomly drawn example from *f*_+_ achieves a higher score than a randomly drawn example from $f_{+}^{\prime }$, as per an optimal score function. Mathematically, for 𝒮 being the family of all real-valued score functions defined on ${\mathbb{R}}^{D}$,

$$\text{AUC}\left(f_{+},f_{+}^{\prime }\right)={\text{max}}_{s\in 𝒮}{\;\text{AUC}}_{s}\left(f_{+},f_{+}^{\prime }\right),$$

where, correcting for ties, ${\text{AUC}}_{s}\left(f_{+},f_{+}^{\prime }\right)=p\left(s\left(X_{f_{+}}\right)>s\left(X_{f_{+}^{\prime }}\right)\right)+\frac{1}{2}p\left(s\left(X_{f_{+}}\right)=s\left(X_{f_{+}^{\prime }}\right)\right)$; $X_{f_{+}}$ and $X_{f_{+}^{\prime }}$ are random variables distributed according to *f*_+_ and $f_{+}^{\prime }$, respectively. Note that AUC is symmetric; i.e., $\text{AUC}\left(f_{+},f_{+}^{\prime }\right)=\text{AUC}\left(f_{+}^{\prime },f_{+}\right)$. It ranges from 0.5 to 1, with a higher value indicating a larger difference between the two distributions and consequently a larger bias. Typically, values between 0.5 and 0.6 are considered to be small enough that the distributions can be interpreted to be practically indistinguishable. A value of 1 corresponds to a perfect classifier; that is, a situation when the supports between $f_{+}^{\prime }$ and *f*_+_ are distinct. Thus, in this work, a value of 0.5 indicates no bias and a value of 1 indicates maximum bias (Fig. 2).

If samples from *f*_+_ and $f_{+}^{\prime }$ were available, AUC(*f*_+_, $f_{+}^{\prime }$) could be estimated by first training a classifier to separate the samples, and then computing AUC in the standard manner as the area under the ROC curve. Though a sample from *f*_+_ is not readily available, such a sample is procured using the approach presented in Methods. The bias between *f*_−_ and $f_{-}^{\prime }$ can be quantified as AUC(*f*_−_, $f_{-}^{\prime }$) and estimated similarly.

## 3. Methods

In order to detect and quantify the bias, we derive an expectation-maximization (EM) algorithm from multi-sample Gaussian mixtures. Under the MB-GC assumptions each of *L*^+^, *L*^−^ and *U* contain examples drawn i.i.d. from a Gaussian mixture. Formally,

$$\forall x\in L^{*},x\sim \sum_{k\in {𝒦}^{*}}v_{k}^{*}\phi_{k}^{*}(x)\;\forall x\in U,x\sim \sum_{k\in {𝒦}^{+}}\alpha w_{k}^{+}\phi_{k}^{+}(x)+\sum_{k\in {𝒦}^{-}}(1-\alpha )w_{k}^{-}\phi_{k}^{-}(x),$$

where the second equation for the distribution of *U* is obtained by combining MB-GC assumptions with Eq. 1 and * is a placeholder for + or −. Note that the resultant distribution is a mixture of *K*^+^ + *K*^−^ components. The combined data log-likelihood is given by

$$ℒ\left(\theta ;L^{+},L^{-},U\right)=\sum_{x\in L^{+}}\text{log}\left(\sum_{k\in {𝒦}^{+}}v_{k}^{+}\phi_{k}^{+}(x)\right)+\sum_{x\in L^{-}}\text{log}\left(\sum_{k\in {𝒦}^{-}}v_{k}^{-}\phi_{k}^{-}(x)\right)+\sum_{x\in U}\text{log}\left(\sum_{k\in {𝒦}^{+}}\alpha w_{k}^{+}\phi_{k}^{+}(x)+\sum_{k\in {𝒦}^{-}}(1-\alpha )w_{k}^{-}\phi_{k}^{-}(x)\right),$$

where ***θ*** = {*α*, ***w***^+^, ***w***^−^, ***v***^+^, ***v***^−^, ***μ***^+^, ***μ***^−^, **Σ**^+^, **Σ**^−^} represent all unknown parameters. To obtain the maximum likelihood estimates of the parameters, we derive the following update equations, under the EM framework.

(EM-update) $$\hat{\alpha }=\frac{1}{\left|U\right|}\sum_{x\in U}\sum_{k\in {𝒦}^{+}}\omega_{k}^{+}(x;\overset{ˇ}{\theta }),\;{\hat{w}}_{k}^{*}=\frac{1}{{\overset{ˇ}{\alpha }}^{*}|U|}\sum_{x\in U}\omega_{k}^{*}(x;\overset{ˇ}{\theta }),\;{\hat{v}}_{k}^{*}=\frac{1}{\left|L^{*}\right|}\sum_{x\in L^{*}}\nu_{k}^{*}(x;\overset{ˇ}{\theta })\;{\hat{\mu }}_{k}^{*}=\frac{\sum_{x\in U}\omega_{k}^{*}(x;\overset{ˇ}{\theta })x+\sum_{x\in L^{*}}\nu_{k}^{*}(x;\overset{ˇ}{\theta })x}{\sum_{x\in U}\omega_{k}^{*}(x;\overset{ˇ}{\theta })+\sum_{x\in L^{*}}\nu_{k}^{*}(x;\overset{ˇ}{\theta })}\;{\hat{\Sigma }}_{k}^{*}=\frac{\sum_{x\in U}\omega_{k}^{*}(x;\overset{ˇ}{\theta })\left(x-{\overset{ˇ}{\mu }}_{k}^{*}\right){\left(x-{\overset{ˇ}{\mu }}_{k}^{*}\right)}^{T}+\sum_{x\in L^{*}}\nu_{k}^{*}(x;\overset{ˇ}{\theta })\left(x-{\overset{ˇ}{\mu }}_{k}^{*}\right){\left(x-{\overset{ˇ}{\mu }}_{k}^{*}\right)}^{T}}{\sum_{x\in U}\omega_{k}^{*}(x;\overset{ˇ}{\theta })+\sum_{x\in L^{*}}\nu_{k}^{*}(x;\overset{ˇ}{\theta })},$$

where ˇ and ˆ are used to represent the current and updated parameters, respectively, during an EM iteration; *α*^+^ = *α* and *α*^−^ = 1 − *α*; $\omega_{k}^{*}(x;\theta )$ is the probability that a given *x* ∈ *U* comes from $\phi_{k}^{*}$; similarly, $\nu_{k}^{*}(x;\theta )$ is the probability that a given *x* ∈ L* comes from $\phi_{k}^{*}$; i.e.,

$$\omega_{k}^{*}(x;\theta )=\frac{\alpha^{*}w_{k}^{*}\phi \left(x;\mu_{k}^{*},\Sigma_{k}^{*}\right)}{\sum_{k\in {𝒦}^{+}}\alpha w_{k}^{+}\phi \left(x;\mu_{k}^{+},\Sigma_{k}^{+}\right)+\sum_{k\in {𝒦}^{-}}(1-\alpha )w_{k}^{-}\phi \left(x;\mu_{k}^{-},\Sigma_{k}^{-}\right)}\;\nu_{k}^{*}(x;\theta )=\frac{v_{k}^{*}\phi \left(x;\mu_{k}^{*},\Sigma_{k}^{*}\right)}{\sum_{k\in {𝒦}^{*}}v_{k}^{*}\phi \left(x;\mu_{k}^{*},\Sigma_{k}^{*}\right)}.$$

Starting with an initial value, as discussed in Section 3.3, the parameters in ***θ*** are iteratively updated using Eq. EM-update until convergence, when the relative change in the log-likelihood, $\left(ℒ\left(\hat{\theta };L^{+},L^{-},U\right)-ℒ\left(\overset{ˇ}{\theta };L^{+},L^{-},U\right)\right)/ℒ\left(\overset{ˇ}{\theta };L^{+},L^{-},U\right)$, is less than a small predefined threshold (*δ*) or until the number of iterations reaches a predefined maximum (*I*).

### 3.1. Estimating Bias

Once ***θ*** is estimated, we use the estimated value of ***w***^+^ to infer the distribution of the unbiased positives, *f*_+_, as per Eq. MB-GC. In order to estimate the bias in the labeled positive sample, we first subsample from *U*, to procure a set, ${\hat{L}}^{+}$, representing estimated *f*_+_. To this end, we use the responsibility, $r^{+}(x;\theta )=\sum_{k\in {𝒦}^{+}}\omega_{k}^{+}(x;\theta )$, giving the probability that a given *x* ∈ *U* is a positive. Precisely, ∀*x* ∈ *U*, if

$$\text{Bernoulli}\left(r^{+}(x;\theta )\right)=\{\begin{array}{ll} 1 & \text{add }x\text{ to }{\hat{L}}^{+},\\ 0 & \text{discard }x, \end{array}$$

where *r*^+^(*x*; *θ*) is used as the success probability of the Bernoulli distribution. Once ${\hat{L}}^{+}$ is procured, we estimate the bias, AUC(*f*_+_, $f_{+}^{\prime }$), by training a classifier between *L*^+^ and ${\hat{L}}^{+}$ treated as positives and negatives, respectively, and compute the AUC using the classifier’s score function. The bias in *L*^−^ can be similarly estimated using the responsibility $r^{-}(x;\theta )=\sum_{k\in {𝒦}^{-}}\omega_{k}^{-}(x;\theta )$ to subsample ${\hat{L}}^{-}$ from *U* and then computing the AUC for a classifier trained to separate ${\hat{L}}^{-}$ and *L*^−^. For a dataset *S* = (*L*^+^, *L*^−^, *U*), we denote the estimated bias as Bias_est_(*S*).

### 3.2. Detecting Bias

We focus the subsequent presentation on bias detection in *L*^+^ only; the detection of bias in *L*^−^ can be approached similarly. Due to model misspecification and errors in the parameter and bias estimation, a bias higher than 0.5 is likely to be estimated, when, in fact, the data is unbiased. To mitigate this issue, we introduce a bias threshold, *τ* ∈ [0.5, 1], and interpret a dataset to contain bias only if its estimated bias is above *τ*. A higher value of *τ* would decrease the probability that an unbiased dataset is detected to have bias (type-1 error), *e*(*τ*). However, it will also decrease the probability that a biased dataset is detected to have bias (power), *q*(*τ*). To achieve a low type-1 error and a high power, we determine an appropriate value of *τ* by controlling for type-1 error on synthetic datasets; see Synthetic Data.

Let ${𝒮}_{\text{syn}}^{\text{ub}}$ and ${𝒮}_{\text{syn}}^{\text{b}}$ be two families of unbiased and biased synthetic datasets, respectively, where each dataset is of the form (*L*^+^, *L*^−^, *U*) and bias is defined as per the current context. Let $e_{\text{syn}}(\tau )=\left|\left\{{\text{Bias}}_{\text{est}}(S)\geq \tau ,S\in {𝒮}_{\text{syn}}^{\text{ub}}\right\}\right|/\left|{𝒮}_{\text{syn}}^{\text{ub}}\right|$ and $q_{\text{syn}}(\tau )=\left|\left\{{\text{Bias}}_{\text{est}}(S)\geq \tau ,S\in {𝒮}_{\text{syn}}^{\text{b}}\right\}\right|/\left|{𝒮}_{\text{syn}}^{\text{b}}\right|$ be the fraction of unbiased and biased synthetic datasets with estimated bias above *τ*, respectively. We define *τ*_*η*_ = min_*τ*_*e*_syn_(*τ*) ≤ *η* as a suitable threshold for which type-1 error computed w.r.t ${𝒮}_{\text{syn}}^{\text{ub}}$ is *η* (typically, *η* ∈ [0, 0.1]); i.e., *e*_syn_(*τ*_*η*_) = *η*. The power computed w.r.t. ${𝒮}_{\text{syn}}^{\text{b}}$ at *τ*_*η*_ is *q*_syn_(*τ*_*η*_). Using this framework, for any real-world dataset *S* = (*L*^+^, *L*^−^, *U*), we enable computing a p-value for bias detection as p-value(*S*) = *e*_syn_(Bias_est_(*S*)), the proportion of unbiased synthetic datasets estimated to have a bias above Bias_est_(*S*).

Note that estimates of type-1 error, power and p-value computed w.r.t. synthetic datasets are representative of their true values to the extent that they capture the diversity of the real-world datasets. In addition to explicitly diversifying the synthetic datasets to a feasible extent, we address this issue by also estimating type-1 error and power w.r.t. selected unbiased and biased real-world datasets, still using the synthetic data threshold; see Data and Results.

### 3.3. Implementation Details

#### Initialization

Parameter estimates of our algorithm are likely sensitive to the initial parameters; it is known to be the case for the standard EM algorithm (GMM) for a single Gaussian mixture sample.^22, 23^ Because we have access to labeled data, we leverage it for parameter initialization. However, in order to introduce more diversity to initialization across multiple restarts, we do not use parameters estimates on only labeled data as our initial parameters; e.g., by using parameters from GMM on each L*. Instead, we initialize parameters in the following steps. (1) Run GMM with K* components on L* to obtain initial estimates of v*, for * ∈ {+, −} and save the location parameter estimates ${\text{u}}^{*}={\left\{u_{k}^{*}\right\}}_{k\in {𝒦}^{*}}$ (2) Run k-means++^24^ on unlabeled data *U* with *K*^+^ + *K*^−^ centers. Sort the centers based on the minimum distance to any location in ***u***^+^. Pick the top *K*^+^ centers to initialize ***μ***^+^ and the remaining centers as ***μ***^−^. (3) Compute the distance from unlabeled points *x* ∈ *U* to each of the *K*^+^ + *K*^−^ centers and assign them to the closest one. This gives an assignment for all points to a cluster which has already been assigned as positive or negative. (4) Use the assignments to compute $\alpha =\frac{\sum_{k\in {𝒦}^{+}}\left|A_{k}^{+}\right|}{\left|U\right|}$, $w_{k}^{*}=\frac{\left|A_{k}^{*}\right|}{\sum_{k\in {𝒦}^{*}}\left|A_{k}^{*}\right|}$ and $\Sigma_{k}^{*}=\frac{1}{\left|A_{k}^{*}\right|}\sum_{x\in A_{k}^{*}}\left(x_{i}-\mu_{k}^{*}\right){\left(x_{i}-\mu_{k}^{*}\right)}^{T}$, where $A_{k}^{+}\left(A_{k}^{-}\right)$ indicate points assigned to the *k*-th positive (negative) cluster.

#### Model Selection

Parameter estimation with EM algorithms when the number of components is unknown is not trivial and many methods exist for model selection.^25, 26^ We employ the one-fold cross-validation-based information criterion (CVIC)^25^ for model selection by running our EM optimization for various values of *K*^+^, *K*^−^ and selecting the model that achieves the highest log-likelihood on a validation set.

#### Hyper-parameters

We assume *K* ≡ *K*^+^ = *K*^−^ for convenience in experimentation. We use the maximum number of iterations *I* = 2000 and the convergence threshold *δ* = 10^−8^ for termination. We run the estimation on each dataset 20 times with different random seeds.

## 4. Data

### 4.1. Synthetic Data

To find appropriate bias thresholds and evaluate our method, we generate synthetic Gaussian mixture datasets, following MB-GC assumptions, from known parameters. This allows us to control bias directly and evaluate performance for different levels of bias in the dataset.

Here *f*_+_ and *f*_−_ are both *K*-component Gaussian mixtures. Their parameters are determined by a given AUC(*f*_+_, *f*_−_) range (e.g., [0.65, 0.7]) and mutual irreducibility parameters, support (*σ* = 0.01) and pairwise responsibility threshold (*ρ* = 0.9), governing the overlap between each pair of components. Let *ϕ*_*i*_ and *ϕ*_*j*_ be two of the 2*K* components and let *Z*_*i*_ and *Z*_*j*_ be samples of 1000 examples each, drawn from *ϕ*_*i*_ and *ϕ*_*j*_, respectively. If more than *σ* fraction of points in *Z*_*i*_ have *ϕ*_*i*_(·) ≥ *ρ*(*ϕ*_*i*_(·) + *ϕ*_*j*_(·)) and, similarly, more than *σ* fraction of points in *Z*_*j*_ have *ϕ*_*j*_(·) ≥ *ρ*(*ϕ*_*i*_(·) + *ϕ*_*j*_(·)), then *ϕ*_*i*_ and *ϕ*_*j*_ are considered to be approximately mutually irreducible.^27^ Starting with random values for the location and shape parameters for each component as well as the mixing proportions ***w***^+^ and ***w***^−^ of the two mixtures (drawn from a flat Dirichlet distribution), the parameters are perturbed until AUC(*f*_+_, *f*_−_), evaluated with $f_{+}(\cdot )/f_{-}(\cdot )$ as the score function (known to be optimal), lies in the desired range and all pairs of the 2*K* components are approximately mutually irreducible w.r.t. *σ* and *ρ*.

We generate 1000 unbiased datasets for each combination of dimensions *D* ∈ {1, 2, 8, 16} and number of components *K* ∈ {2, 4, 8}. The class prior *α* is sampled uniformly from the range [0.01, 0.99] for each dataset. Seven AUC(*f*_+_, *f*_−_) ranges, [0.65, 0.7], [0.7, 0.75], …, [0.95, 1] are approximately equally represented in the 1000 datasets for each setting. For the unbiased datasets, $f_{+}^{\prime }$ and $f_{-}^{\prime }$ are set equal to *f*_+_ and *f*_−_, respectively.

To evaluate performance of bias estimation against known values of bias, we generate 1750 datasets for each dimension and number of components for varying levels of bias AUC(*f*_+_, $f_{+}^{\prime }$) between 0.5 and 1 (Fig. 2b). First *α*, *f*_+_ and *f*_−_ are generated as for the unbiased data, where the seven AUC(*f*_+_, *f*_−_) ranges are equally represented across the 1750 datasets. A desired range of bias is achieved by drawing random mixing proportions, ***v***^+^, from a flat Dirichlet distribution until AUC(*f*_+_, $f_{+}^{\prime }$) computed with the optimal score function f+(⋅)f+′(⋅) is in the target bias range. The five bias ranges [0.5, 0.6], [0.6, 0.7], …, [0.9, 1] are equally represented across the datasets. For simplicity, $f_{-}^{\prime }$ is set equal to *f*_−_.

Each dataset has 100, 000 unlabeled points from *f* = *αf*_+_+(1−*α*)*f*_−_ and 5, 000 labeled points from each $f_{+}^{\prime }$ and $f_{-}^{\prime }$ with the chosen parameters. Figure 2a shows examples of 1D distributions for different values of AUC(*f*_+_, *f*_−_) within the range we use to sample synthetic data. These examples illustrate the complexity of synthetic datasets; even for higher AUC(*f*_+_, *f*_−_), the positive and negative distributions are not easily distinguished.

### 4.2. Biomedical Data

We selected 8 biomedical datasets from the the UCI Machine Learning Repository^28^ to apply our methods. The following datasets were used, with a note that for each we give the number of examples, the fraction of examples from the positive class (*α*) and the number of features *D* in parentheses: Activity recognition with healthy older people using a wearable sensor^29^ (52481, 0.29, 8), Epileptic Seizure Recognition^30^ (11500, 0.18, 178), Smartphone-Based Recognition of Human Activities and Postural Transitions^31^ (10929, 0.16, 561), Mushroom^28^ (8124, 0.21, 126), HIV-1 protease cleavage^32^ (6590, 0.20, 160), Splice-junction Gene Sequences^33^ (3190, 0.24, 287), Parkinsons Telemonitoring^34^ (5875, 0.48, 20), and Physicochemical Properties of Protein Tertiary Structure^28^ (45730, 0.13, 9).

**Fig. 3:**
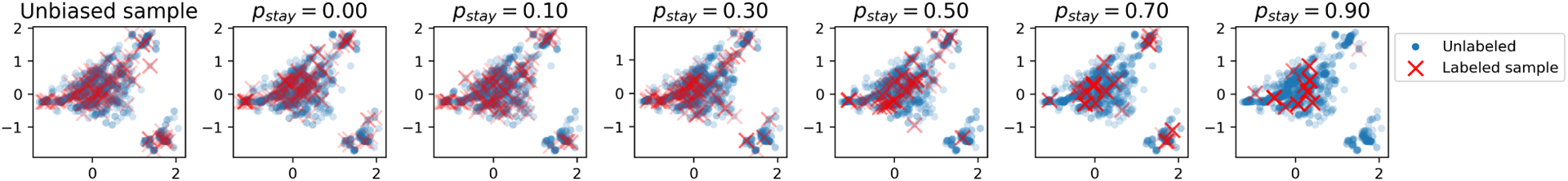
Unbiased (far left) and biased samples from the dataset HIV^32^ with varying probability of resampling a point *p*_stay_. Features are illustrated projected onto the first two principal components.

Datasets were constructed by assigning one class as positive and the remaining as negative for multi-class data or setting a threshold for regression data. For each problem, 100 unbiased datasets were generated by selecting a subset of labeled points uniformly. We generate 250 biased datasets for each biological dataset through Markov sampling. First a point *x*_*i*_ is selected uniformly at random from the positive class. The same point is resampled with some probability *p*_stay_ and a new point *x*_*j*_ is selected with probability 1 − *p*_stay_. The transition probability Pr(*x*_*j*_|*x*_*i*_) is proportional to the inverse of the squared Euclidean distance between points ∥*x*_*i*_ − *x*_*j*_∥^2^. Since the true bias cannot be measured directly, we use the probability of resampling *p*_stay_ as a proxy for bias. Higher values of *p*_stay_ correspond to higher bias in labeled data since the feature space will be less uniformly sampled (Fig. 3). In each case, 20% of points are held out as a validation set used for model selection. We reduce the dimensionality with PCA for datasets with more than 8 features.

## 5. Experiments

### Empirical Null Distribution and Bias Threshold

We use synthetic Gaussian mixture datasets to determine the bias threshold for a range of dimensions *D* ∈ {1, 2, 8, 16} and number of components *K* ∈ {2, 4, 8}. We consider bias in positive class, but the method for estimating bias in the negative class or both would follow the same process. We run the EM optimization on each unbiased dataset to estimate all unknown parameters, ***θ***. We use the estimated parameters $\hat{\theta }$ to compute the estimated bias for the positive class, AUC($\hat{f_{+}}$, $\hat{f_{+}^{\prime }}$), where $\hat{\cdot }$ indicates the parameters estimated by the optimization procedure and the distributions parameterized by them. The true bias AUC(*f*_+_, $f_{+}^{\prime }$) for these datasets is exactly 0.5 since the distributions are identical (no bias), but because there is error in the estimation $\hat{\theta }$, AUC($\hat{f_{+}}$, $\hat{f_{+}^{\prime }}$) ≥ 0.5. For each setting of dimension *D* and number of components *K* used to generate the datasets, we determine *τ*_*η*_(*D*, *K*) for *η* ∈ {0.05, 0.10} of datasets with AUC($\hat{f_{+}}$, $\hat{f_{+}^{\prime }}$) ≥ *τ*_*η*_(*D*, *K*).

### Model Selection

To apply the appropriate bias threshold *τ*_*η*_(*D*, *K*) to any data it is important to know the number of components that best represent the data and use the threshold found for that setting (dimension is known). However, the true or best value of *K* is not generally known for any dataset. We evaluate the effect of unknown *K* for finding the threshold *τ*_*η*_ by running the optimization on unbiased datasets for *K* ∈ {2, 4, 8} on all datasets, regardless of which value was used to generate the data. For each dataset, we compute the estimated parameters log-likelihood on a validation set and choose the model that maximizes the value. The validation set is generated with the same parameters as the original dataset.

### Bias Quantification and Detection

To evaluate our method in detecting and estimating bias, we run our EM optimization algorithm on synthetic and biological datasets with varying amount of bias and report the estimated bias. For synthetic data where the true bias is known, we evaluate power for each level of type-1 error, *η* ∈ {0.05, 0.10}. Ground truth biased datasets 𝓑 are those where the true bias AUC(*f*_+_, $f_{+}^{\prime }$) > 0.5, for *K* number of components. Predicted biased datasets $\hat{𝓑}$ are those where $\text{AUC}\left(\hat{f_{+}},\hat{f_{+}^{\prime }}\right)\geq \tau_{\eta }(D,\hat{K})$ for $\hat{K}$ selected through model selection. Power is estimated as q(τ)=|𝓑^||𝓑|.

## 6. Results and Discussion

**Fig. 4:**
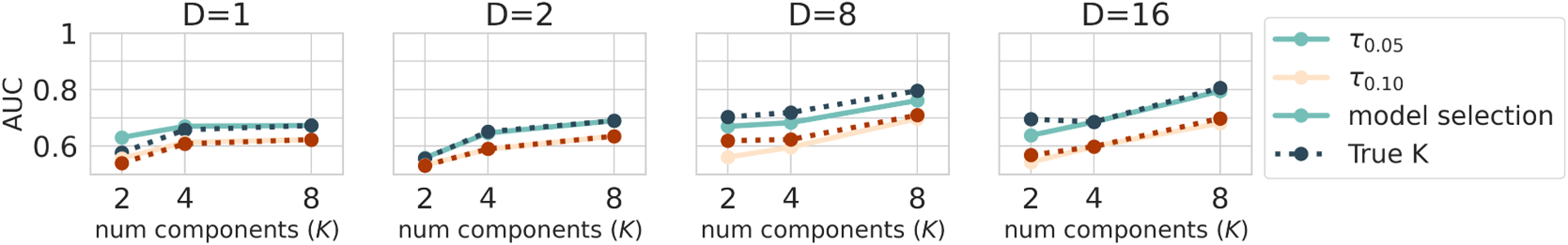
Bias (AUC(*f*_+_, $f_{+}^{\prime }$)) thresholds found from parameter estimation on unbiased data sets.

Figure 4 illustrates the thresholds found for each dimension and number of components. When the number of components, *K*, is smaller, parameter estimation more reliably estimates the bias lower. As the number of dimensions and number of components increases, so does the complexity of the optimization problem and the estimated value of bias. These results suggest the utility of finding dimension- and component-specific thresholds, and the empirical null distribution for ascertaining bias.

**Fig. 5:**
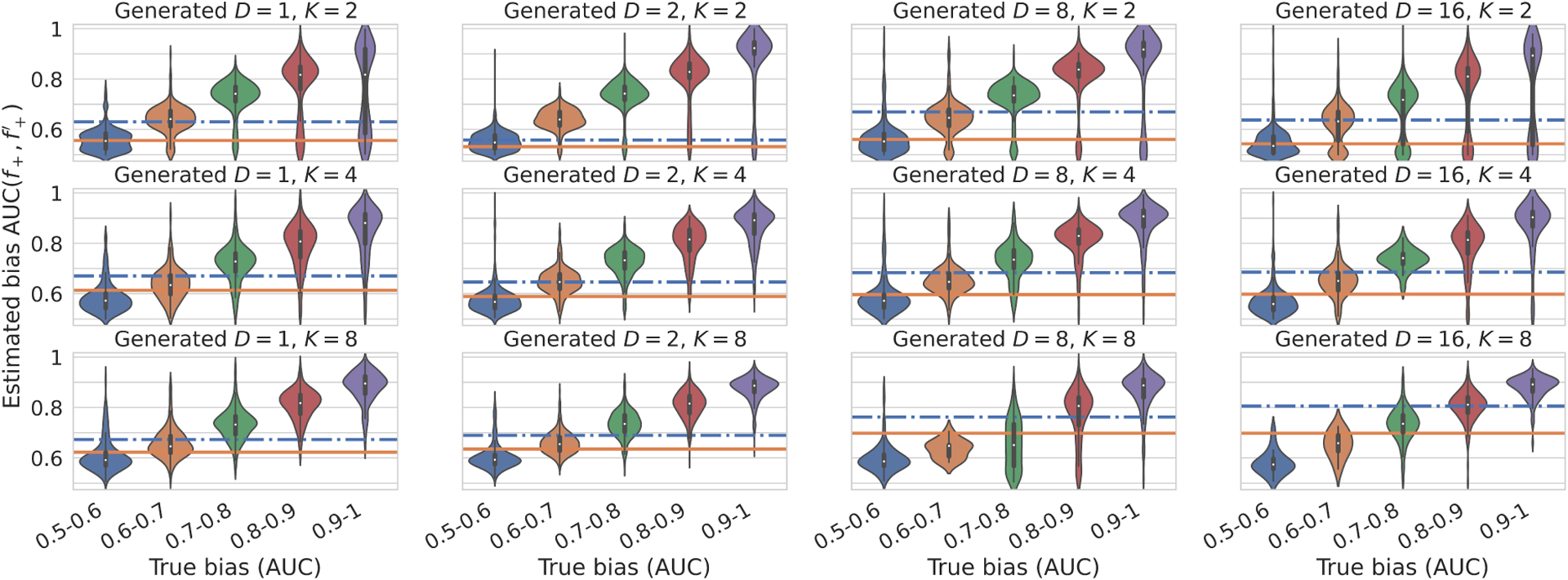
Estimated bias on Gaussian mixtures with varying true bias AUC(*f*_+_, $f_{+}^{\prime }$). Bias thresholds *τ*_0.05_, *τ*_0.10_ shown as dash-dotted and solid lines, respectively.

**Fig. 6:**
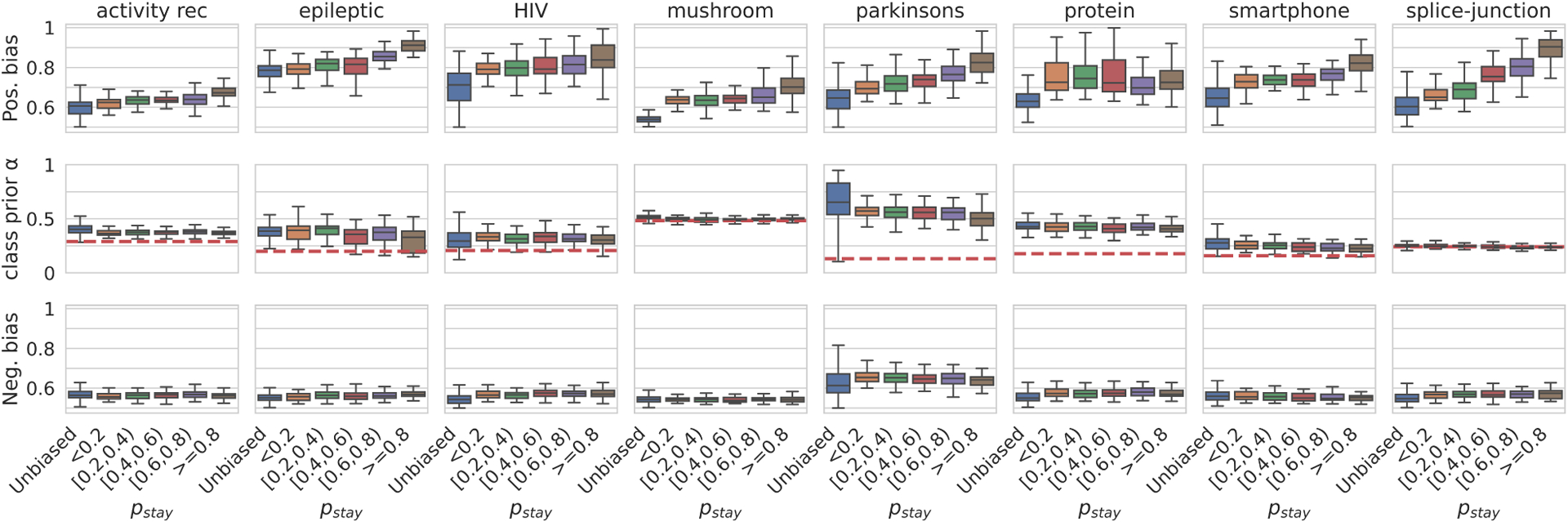
Bias and parameter estimation for biomedical datasets. Each column shows results for samples from each dataset. Top: Bias estimation for positive class for unbiased (leftmost) and biased sampled datasets for increasing levels of *p*_stay_, corresponding to larger bias. Middle: Estimation of the class prior *α* with true value shown as dashed line. Bottom: Bias estimation for negative class, which is unbiased in each case.

**Fig. 7:**
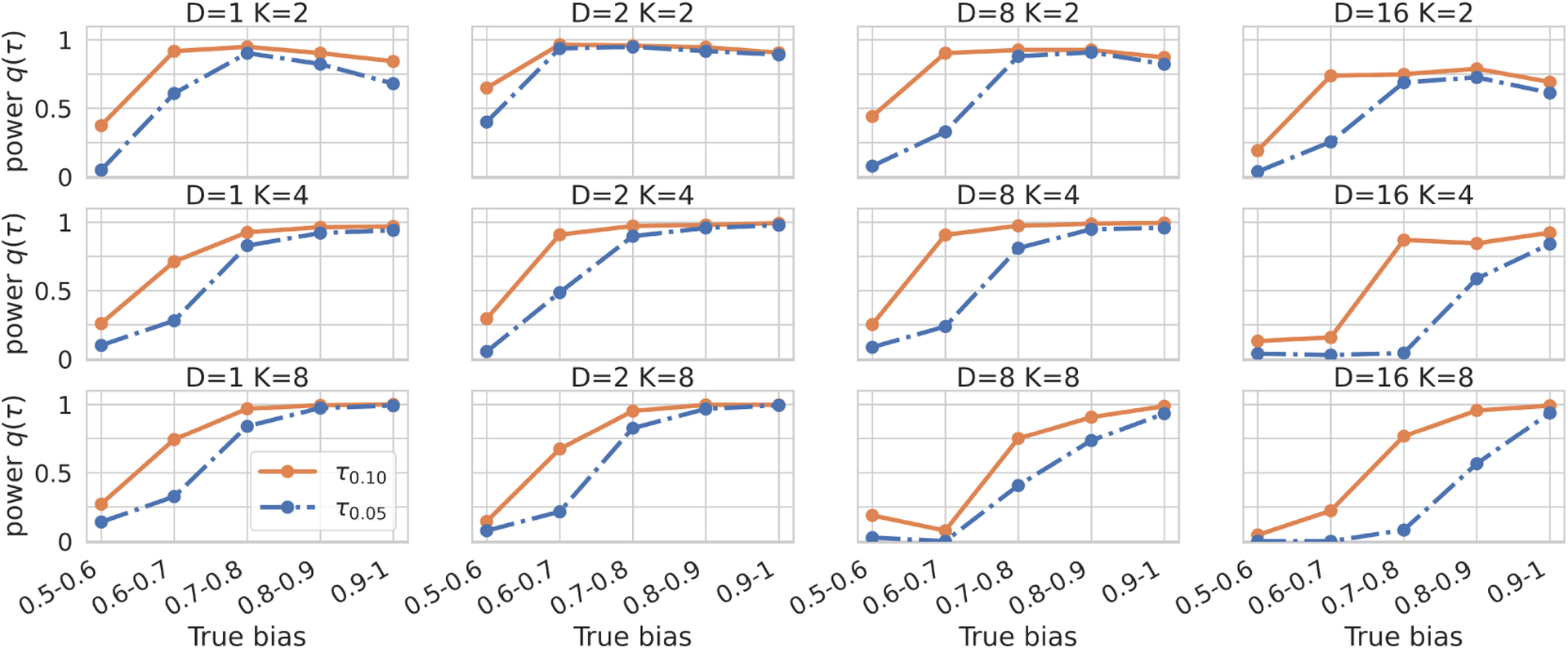
Power *q*(*τ*) of bias prediction for type-1 error *η* ∈ {0.05, 0.10} on unbiased datasets.

Results on quantification of bias on synthetic (Fig. 5) and biomedical (Fig. 6) data show increasing estimated bias as true bias increases. Note that for biomedical datasets the true bias is unknown and *p*_stay_ is not a direct measurement of bias; different data sets have different levels of compactness in their feature space. Since the sampling probability is proportional to the inverse distance between points, the bias is also dependent on the density of points. Bias will differ across datasets for the same value of *p*_stay_ and estimated bias cannot be directly compared between datasets. However, for each datasets bias should increase as the sampling less uniform, *i*.*e*. *p*_stay_ increases. In synthetic data, we see excellent power (Fig. 7) for the type-1 error of 0.05 across all levels of bias, dimensionality *D* and the number of components (*K*) per class-conditional distribution. We also see for high-bias datasets (AUC(*f*_+_, $f_{+}^{\prime }$) ≥ 0.9) on datasets with two components, that some datasets have a low estimated bias. Our investigation showed that to generate datasets with high bias and few components, the mixing proportions $w_{k}^{+}$ or $v_{k}^{+}$ must be very skewed, making the optimization difficult, sometimes unrealistically so. For one dimension, the average minimum value of the smallest $w_{k}^{+}$ for datasets with AUC(*f*_+_, $f_{+}^{\prime }$) ≥ 0.9 is 0.01, 0.07 for 0.8 ≤ AUC(*f*_+_, $f_{+}^{\prime }$) < 0.9, and 0.19–0.23 for AUC(*f*_+_, $f_{+}^{\prime }$) < 0.8.

Figure 7 shows the power for bias detection on synthetic datasets for type-1 error *η* ∈ {0.05, 0.10}. For each setting we see generally higher power in bias detection as the true bias increases. For higher type-1 error, the detection achieves a higher power. Again there is a drop in performance for *K* = 2 in high-bias datasets due to the challenging nature of these datasets.

For real datasets we also show that our estimation of *α* and negative bias is not generally affected by increasingly biased samples of the positive class (Fig. 6, middle and bottom rows, respectively). Our EM algorithm is still able to detect that the set of unbiased labels from the negative class are truly unbiased (a low value of AUC($\hat{f_{-}}$, $\hat{f_{-}^{\prime }}$)). The estimation for bias for negative class in UCI results is consistently better than the estimation of bias for unbiased positive samples because *α* is always less than or equal to 0.5. Higher estimated bias in negatives seems to be correlated with overestimation of the class prior *α*, particularly exemplified in the parkinsons dataset.

## 7. Conclusion

Despite a broad awareness that biased data may adversely impact the deployment of machine learning tools in biomedicine, there is a surprising dearth of methods built to ascertain the existence and the level of bias in available data. We set out to address this deficiency by developing and extensively evaluating a bias estimation method based on reasonable assumptions. We used synthetic and real-world biomedical data to show that technologies for bias detection and ultimately correction can be realistically implemented in future data processing pipelines.
